# Taquicardia Atrial Para-Hissiana e Taquicardia de Reentrada Nodal Atrioventricular: 25 Anos Depois, a Mesma História?

**DOI:** 10.36660/abc.20201149

**Published:** 2021-01-27

**Authors:** Mauro Toniolo

**Affiliations:** Divisão de Cardiologia Hospital Universitário “S. Maria della Misericordia” Udine Itália Divisão de Cardiologia, Hospital Universitário “S. Maria della Misericordia”, Udine - Itália

**Keywords:** Seio Não Coronário de Valsalva, Taquicardia Atrial, Taquicardia de reentrada nodal atrioventricular

A história recente da taquicardia atrial para-Hissiana (TAPH) se parece muito com a história mais antiga da taquicardia de reentrada nodal atrioventricular (TRNAV). O debate sobre os limites anatômicos precisos da reentrada nodal atrioventricular continua hoje no que diz respeito à TRNAV,^1^ mesmo 75 anos após a primeira suspeita de que alguns mecanismos de taquicardia supraventricular poderiam envolver a região do nó atrioventricular.^2^ Nesse tipo de arritmia, o primeiro alvo para a terapia ablativa por cateter foi a via rápida do nó atrioventricular:^3^ essa abordagem apresentou alta taxa de sucesso, mas a indução do bloqueio atrioventricular foi encontrada em mais de um a cada cinco pacientes.^4^ Após alguns anos, foi proposta uma abordagem por via lenta,^5^ que se mostrou mais eficaz e segura do que a abordagem por via rápida.^4^ Por alguns anos, essa nova abordagem gerou algum debate na comunidade de eletrofisiologia. Alguns autores chegam a sugerir a substituição de uma técnica por outra, desde que a TRNAV persistisse.^6^ Atualmente, a abordagem pela via rápida foi definitivamente abandonada e, quando pensamos em ablação por cateter de TRNAV, pensamos apenas na ablação por via lenta.

A TAPH é um grupo de taquicardias atriais (ATs) com origem próxima à região do feixe de His. Sua prevalência é bastante elevada em algumas casuísticas,^7^ por isso é importante aprender a reconhecê-las e tratá-las. Hoje em dia, da mesma forma que a TRNAV, também para a TAPH existe um debate tanto sobre o sítio anatômico de origem desse tipo de arritmia,^8^ tanto sobre o mecanismo^9 , 10^ quanto sobre a melhor abordagem de ablação por cateter.^11 - 14^ Alguns autores sugerem a presença de um pequeno circuito reentrante adjacente ao anel tricúspide;^10^ outros descreveram a TAPH como ATs focais originando-se de vários pontos ao redor do anel tricúspide ou mitral.^9^ As TAPH são passíveis de ablação por cateter por várias abordagens, incluindo o septo interatrial direito, septo interatrial esquerdo por punção transeptal e seio não coronário (SNC) de Valsalva da raiz aórtica por abordagem transaórtica via artéria femoral.

Nesta edição dos Arquivos Brasileiros de Cardiologia, Chokr et al.^15^, é importante descrever uma série de casos de pacientes submetidos à ablação do seio de Valsalva. Um dos achados mais relevantes de seu trabalho foi que esse tipo de ablação é viável com uma exposição radiológica relativamente baixa, também sem um sistema de mapeamento eletroanatômico 3-D e sem a ecocardiografia intracardíaca, método que a comunidade de eletrofisiologia atual parece não conseguir deixar de fora. Por esse motivo, o método pode ser viável em todos os laboratórios de eletrofisiologia.

No entanto, mesmo que já tenha sido comprovado que uma abordagem ablativa do seio de Valsalva é mais eficaz e segura do que uma abordagem da região do feixe de His no septo interatrial direito e/ou esquerdo,^12 , 14^ independentemente do local de ativação atrial mais precoce, alguns autores sugerem como primeira escolha em algumas situações a ablação do septo interatrial direito ou esquerdo,^11 , 13^ apesar do risco de danos ao sistema de condução atrioventricular. Provavelmente, como na história do TRNAV, daqui a mais 25 anos, quando pensarmos em ablação para TAPH, pensaremos apenas na abordagem do seio de Valsalva, e a abordagem da região do feixe de His será definitivamente esquecida!
