# Supplementary material for: Taquicardia Atrial Para-Hissiana e Taquicardia de Reentrada Nodal Atrioventricular: 25 Anos Depois, a Mesma História?
Source: Arq Bras Cardiol. 2021 Jan 27;116(1):127–8. [Article in Portuguese] doi: 10.36660/abc.20201149 (PMC8159513; doi:10.36660/abc.20201149)
Supplement: Translation

# Translation: Para-Hisian Atrial Tachycardia and Atrioventricular Nodal Reentry Tachycardia: After 25 Years The Same History?

**Authors:** Mauro Toniolo

**Affiliations:** Italy Cardiology Division, University Hospital “S.Maria della Misericordia”,1 Udine - Italy

**Keywords:** Non Coronary Sinus of Valsalva, Atrial Tachycardia, Atrioventricular Nodal Reentry Tachycardia

The recent history of the para-hisian atrial tachycardia (PHAT) looks a lot like the older history of the atrioventricular (AV) nodal reentry tachycardia (AVNRT). The debate about precise anatomic boundaries of AV nodal reentry continues today again in the AVNRT,^1^even 75 odd years after the first suspect that some mechanisms of supraventricular tachycardia could involve the region of the AV node.^2^In this type of arrhythmia, the first target for the catheter ablative therapy was the fast pathway of the AV node:^3^this approach showed a high success rate, but induction of AV block was found in more than one every five patients.^4^After a few years, an approach was proposed from the slow pathway,^5^that proved to be more effective and safer than the approach from the fast pathway.^4^For some years, this new approach generated some debate in the electrophysiology (EP) community. Some authors even suggested to cross over from one technique to the other as long as AVNRT persisted.^6^Nowadays, the approach from fast pathway is definitely abandoned and when we think of catheter ablation of AVNRT, we think only about ablation of the slow pathway.

PHAT are a group of atrial tachycardia (ATs) originating near the His-bundle (HB) region. Their prevalence is quite high in some casuistries,^7^therefore it is important learning to recognize and to treat them. Nowadays, in the same manner of AVNRT, also for PHAT a debate is present for both the anatomic site of origin of this type of arrhythmia,^8^both about the mechanism,^9 , 10^both about the best catheter ablation approach.^11 - 14^Some authors hypothesized the presence of a small re-entrant circuit adjacent to the tricuspid annulus;^10^in contrast, other authors described PHAT as focal ATs arising from various location around the tricuspid or mitral annuli.^9^PHAT are amenable to catheter ablation from multiple approaches including right inter-atrial septum, left inter-atrial septum by a transeptal puncture and non coronary sinus (NCS) of Valsalva of the aortic root by a transaortic approach via the femoral artery.

In this issue of *Arquivos Brasileiros de Cardiologia* , Chokr et al.^15^importantly, describe a case-series of patients ablated from the NCS of Valsalva. One of the most relevant findings of their work is the fact the this type of ablation is feasible with a relatively low radiological exposure also without a 3-D electroanatomic mapping system and without the intracardiac echocardiography, of which the current EP community seems to be unable to do without. For this reason, this method can be feasible in every EP lab. However, even if it was already proved that an ablative approach from the NCS of Valsalva is more effective and safer than an approach from the HB region in the right and/or left inter-atrial septum,^12 , 14^regardless of the earliest atrial activation site, there are authors that in some situations suggest as first choice the ablation of the right or left inter-atrial septum,^11 , 13^despite the risk of creating damages to the AV conduction system. Probably, as in AVNRT history, in another 25 years, when we will think about PHAT ablation, we will think only about the NCS of Valsalva approach, and the approach from the HB region will be definitely forgotten!
